# Student Perceptions of the Resilience in a Confinement Due to COVID-19 in University of A Coruña: A Qualitative Research

**DOI:** 10.3390/bs12080294

**Published:** 2022-08-19

**Authors:** María-Paula Ríos-de-Deus, María-Luisa Rodicio-García, Laura Rego-Agraso, María-José Mosquera-González, Marta Elena Losa-Iglesias, Ricardo Becerro-de-Bengoa-Vallejo, Daniel López-López

**Affiliations:** 1 Department of Specific Didactics, Research, and Diagnose Methods, Grupo de Investigación FORVI (Formación y Orientación para la Vida), Universidade da Coruña, 15071 A Coruna, Spain; 2 Department of Physical and Sports Education, Grupo de Investigación FORVI (Formación y Orientación para la Vida), Universidade da Coruña, 15179 A Coruna, Spain; 3 Faculty of Health Sciences, Universidad Rey Juan Carlos, 28943 Alcorcón, Spain; 4 School of Nursing, Physiotherapy and Podiatry, Universidad Complutense de Madrid, 28040 Madrid, Spain; 5 Research, Health and Podiatry Group, Department of Health Sciences, Faculty of Nursing and Podiatry, Industrial Campus of Ferrol, Universidade da Coruña, 15403 A Coruna, Spain

**Keywords:** resilience, wellness, personal growth, qualitative research, COVID-19, higher education

## Abstract

The home confinement caused by COVID-19 has caused university students to express feelings, negative experiences, and concerns about the confinement situation they were experiencing. This prompted the development of research on resilience, which shows that it is closely related to well-being. The general objective is to determine if resilience acts as a guarantor of personal growth and, therefore, of the self-perception of well-being. The research is developed with qualitative methodology and is framed in the interpretative phenomenological analysis approach and is framed in the hermeneutic-dialectical method. The selection of participants was carried out through an intentional sampling, by non-random methods, among university students. Fifty-two students participated, 41 are women (78.84%) and 11 men (21.15%), with an average age of 20.7 years. The information was collected through a diary card in which they had to collect their experiences and prepare a short speech identifying three temporal moments of confinement: beginning, during, and end. The information was collected between 16 April and 15 May 2020. An inductive analysis was carried out, and the emerging categories were defined: personal growth, resilience, and well-being. Personal growth materializes through three subcategories: personal changes, interpersonal changes, and changes in the philosophy of life. The university students showed that the vital transformation related with resilience acts as a guarantor of personal growth and self-perception of well-being. A voluntary, conscious, and intelligent evolution of people is detected, and personal changes, interpersonal changes, and changes in the philosophy of life are identified as factors of personal growth.

## 1. Introduction

On 14 March 2020, the legal regulations to manage the health crisis caused by COVID-19 were published in Spain. Among the measures planned to protect health, a home confinement was decreed, which led, among other measures, to the suspension of face-to-face educational activity, including university teaching.

This situation has promoted the development of multiple investigations in the university context; however, all are quantitative in nature and are related to the ability of people to adapt to adverse situations and study the relationships of resilience with psychological, physical, and social impacts on daily life [1]. Some value the way in which the university community develops strategies to adapt to the demands of virtual education [2], confirming the existence of a digital divide [3], and others analyze the confinement among university students, considering life satisfaction, resilience, and social capital [4].

Resilience does not have a single accepted definition [5,6], although there is consensus in its consideration as an effective defense mechanism and personal and social improvement against adverse situations [7,8]. Thus, the diversity of the scientific evidence on this concept allows us to conclude that people with a resilient personality face difficulties with greater success than those who do not [9]. For this reason, it can be affirmed that it is a holistic concept that, from different research areas, explores the adaptation capacity of the human being at a personal, social, and family level [10].

It has been proven that people with the capacity and flexibility to adapt to the changes experienced throughout their lives deal constructively and positively with these adverse situations, which causes them to maintain a satisfactory personal balance, because of a learning process. Therefore, the resilience capacity helps to improve and facilitates the discovery and use of personal strengths, being relevant in this process, particularly in the interrelations of people who share similar conditions and/or lifestyles [11].

Research on the self-perception of well-being, carried out in the field of the social sciences, has developed different theoretical models. There are authors who consider (for example, Ryff and Keyes) [12] that it is achieved through the self-regulation of behavior, defining as dimensions of their study such as self-control, life purpose, self-esteem, self-knowledge, and positive relationships with other people. On the other hand, others relate it to positive emotional associations, which cause an increase in awareness and help to understand the real situation that is being experienced; this increases emotional well-being [13]. Moreover Seligman states that people who do not give up and face difficulties with optimism achieve well-being and manage to overcome difficult experiences quickly [14].

It can be affirmed, therefore, that resilience and well-being are concepts that are closely related, and this is demonstrated in the studies by those who point out the existence of a close link between the two; this relationship is considered positive and significant for the effective resolution of problems [15].

Research by Calhoun and Tedeschi confirms that the majority of people who have faced adverse situations have achieved personal growth reaching well-being through three categories [16]: personal changes [17], changes in interpersonal relationships [18], and changes in the philosophy of life [19] and when there is a balance between the actions taken to deal with problematic situations and the well-being caused by overcoming the situation. These actions are aimed at development, evolution, and personal and interpersonal growth, as well as causing changes in the way of thinking and acting (philosophy of life) [20].

There is sufficient evidence substantiating the association of resilience with well-being [15] obtained in extreme situations or traumatic events throughout the life cycle of people [16,21]; however, during the confinement situation, research confirms the increase in academic stress in university students [22,23]. This reality was evidenced in the students of the Social Education Degree of the Faculty of Educational Sciences of the University of A Coruña (UDC), and was chosen because it is the context in which the authors develop their teaching and research activity and where they have access to the population under study.

These students expressed feelings, negative experiences, and concerns about the discomfort they felt and the maladjustment they were experiencing.

Given this reality, this research work was proposed with the aim of determining whether the rapid, voluntary, conscious, and intelligent adaptation (resilience) of university students to the situation of home confinement caused by COVID-19 acts as a guarantor of personal growth and of the self-perception of well-being.

## 2. Materials and Methods

### 2.1. Design and Sample

A qualitative methodology was used to allow participants to describe behaviors, thoughts, and personal feelings in their own words without limitation, and thus to collect information [24] about the particularities of each person in a situation never experienced or imagined in the world. Moreover, our investigation followed criteria according to the Consensus-Based Checklist for Reporting of Survey Studies (CROSS) [25].

This study is part of a narrative investigation aimed at obtaining information on the experiences, feelings, and emotions of students in order to obtain their perceptions [26] regarding the impact of confinement on their personal adaptation. It is part of the interpretive phenomenological analysis approach, since it seeks to understand the meanings associated with the experience lived by the participants [27], and is part of the hermeneutical-dialectical method because the intentional meaning of the behavior of the participants is embodied through his writings (Cerrón [28], and was completed between 16 April 2020 and 15 May 2020.

The selection of the participants was carried out through intentional sampling by non-random methods. The target population was the student body of the Degree in Social Education at the University of A Coruña (UDC), which was enrolled in the subject of Evaluation Methods for Socio-educational Programs and Services during the 2019/20 academic year; subject taught by one of the researchers. The inclusion criterion was being aged 18 or older and having provided informed consent to participate. The exclusion criterion was a refusal to sign the consent form or an inability to understand the instructions necessary to carry out the present study.

### 2.2. Procedure

A diary file [29] was used for data collection, which was prepared ad hoc following a standard diary template [30]. The students were asked to record their experiences, behaviors, and emotions, stating the date of each entry.

The documents prepared by the students form a social construction [17] from the confinement context. It is a process built and written by people within a particular reality never experienced.

Each example given by students has a purpose in a particular context and is intended for a specific audience: researchers [31]. In this framework, a content analysis is performed. The documents prepared by the students form a social construction [29] from the confinement context. It is a process built and written by people within a particular reality never experienced.

The people who narrated their experience were part of the class group of one of the research teams and included those who voluntarily wanted to participate; moreover, these the only people who had access to the information collection record-diary.

Information was provided through the Virtual Campus of the UDC and the Microsoft Teams platform (means enabled by the University for communication between the university community) about the nature and methodology of the research. Once the interest in participating was expressed, they were given the informed consent that they had to return covered and signed.

### 2.3. Ethics Considerations

Ethical principles and fundamental rights were respected, and the results were treated with the confidentiality determined by current regulations. For this reason, each participant was assigned an alphanumeric code, whose correspondence with the person is only known by the researchers. The letters (I, D, or F) correspond to the start, during, and end of the indicated period, and the number was assigned consecutively.

### 2.4. Sample Size Calculation

We used the software G * Power 3.1.9.2 (G * Power ©; University of Düsseldorf; Düsseldorf, Germany) to calculate the sample size to observe differences before and after the intervention study with a statistical confidence of 95%. Therefore, a 2-tailed hypothesis test and a large effect size of 0.90, an α-error of 0.05, and a power of analysis of 0.80 (β error = 20%) were selected. The result obtained was 18 participants. Considering the possibility of loss to follow-up, a total of 52 participants were recruited.

### 2.5. Statistical Analysis

The student’s writings were analyzed with the MAXQDA 2020 version 20.2.0 program.

The principles of hermeneutic triangulation [32] were used, selecting the appropriate and significant information in an organized manner. An inductive analysis was carried out, and logically structured concept classifications were established, from which the emerging categories and subcategories were defined. This made it possible to interpret the content of the discourse and group-related ideas using one or two words as a unifying criterion [33]. Next, experts in qualitative methodology were required to carry out the analysis of the information to corroborate and verify the results; for this, they were provided with the literal transcription of the writings and the categories and subcategories obtained, along with their definition and textual examples.

This triangulation process made it possible to contrast information from different sources and ensure that the sampling is sufficient due to saturation and because there is no new information. This made it possible to guarantee that implementing a qualitative methodology does not have to be at odds with scientific rigor because the results have been validated and refuted, taking into account certain considerations that provide validity and reliability to the research.

**Figure 1:**
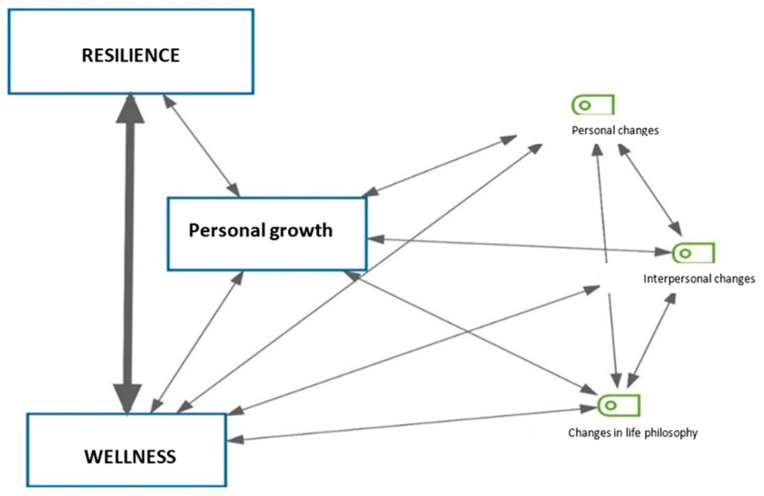
Map of categories.

Once the coincidence between both analyses has been verified, the three main categories that emerge from the discourses are: personal growth, resilience, and well-being. Personal growth materializes through three subcategories: personal changes, interpersonal changes, and changes in the philosophy of life (Figure 1).

## 3. Results

In total, 52 students participated, which implies 64.19% of the total number of students enrolled in the subject, of which 41 are women (78.84%) and 11 are men (21.15%) and have a mean age of 20.7 years.

The fact that the number of women is higher is due to the nature of the female career that a degree in social education is associated with, in which the largest number of students who enroll are women.

The analysis of the relationship between the categories and the frequency of words is detailed in Table 1. The higher the percentage, the higher the frequency of the repetition of the category in the discourse.

The vital transformation of the students is perceived through the evolution of the abilities manifested in each of the categories, in which their evolution and personal growth was evident as the period of home confinement progressed.

To show this, the most significant textual examples of each of them have been collected and written in italics. The three essential moments of the information collection period are identified with the letters and figures indicated above.

Personal growth is presented as a voluntary and intelligent renewal and materializes in the changes derived from the appearance and/or improvement of personal skills such as self-esteem, self-knowledge, self-control, perseverance, feelings and self-care, which are testimony of the change in the ways of perceiving, feeling, and interpreting reality.

Self-esteem. I am very scared and afraid [I9]; I thought I wouldn’t be able to get over it, but little by little I’m seeing that it’s not so bad at home [D27]; I have been able to meet this challenge and overcome it [F1].

- Self-knowledge: I think it will be a good time to reflect and get to know myself [I12]; It is helping me to change and get to know myself [D2]; I have learned to love myself, now I trust what I do […] [F15]; It helped me to change and get to know myself [F2].
- Self-motivation: I think I am strong [I10]; Despite the fact that the environment in the country is beginning to be worrying, I am calm and I feel strong for what is to come [I7]; I coped with the [F2] situation.
- Self-control: I have noticed a change in my mood and feelings as the days of confinement passed [D11]; The situation improves for me, I learn to live with it and I am calmer [F41].
- Perseverance: At first it seemed like I was on vacation [I23]; […] after 15 days I have set myself some goals [D1]; In difficult moments, be persistent [D18].
- Feelings: Uncertainty and I feel scared [I7]; I feel lonely [I15]; Impotence for so many sick people [I23]; Fear for all the infected people and not knowing when this will end [D17]; […] anguish, sadness [D31]; Fear, nervousness and fear of contagion [D51].
- Self-care: In the first 15 days of confinement I have gained weight, I do nothing but eat [I38]; I realize that I have to take care of myself, […] [I48]; After a month of confinement, I have decided to set myself some schedules and guidelines to organize the days and be able to make the most of them [D9]; Think about what my priorities are, I take care of myself [D19]; I start exercising to keep my body and mind healthy [D17].

The development of relational skills is shown through the expression of the changes manifested in relationships with family and friends. In some cases, due to physical distance, these relationships have disappeared (especially with some friends) and, in others, the channel of communication has been changed, with social networks being an important point of support.

- Family relationships: I spend more and more time talking with the people at home, something I haven’t experienced for a long time [I12]; In my case, this moment is helping me to value my family and friends, who, despite the distance, I know I can count on them when I need it and have them close, in addition to questioning what is truly important in life [D7].
- Relationships with friends: I realize the friends that really matter to me and with whom I want to be [D21]; My group of friends has been reduced [F28].
- Social networks: I think much more about my loved ones, friends and family and I make video calls with them [D51]; In general, my activity in social networks increased considerably; as well as group video calls with friends [F4]; I have coped with the situation thanks to the fact that I have kept in touch with family and friends through video calls and messages on social networks [F33].

The change in the fundamental principles and ideas that govern the philosophy of life of the participants is reflected in the self-perceived development of solidarity, empathy, responsibility, and that they have been able to value those things that they previously regarded as normal, to which they did not give importance and passed them totally unnoticed.

- Solidarity: I think of ways to help the families that are most disadvantaged after this situation [D26]; The situation has led me to be more supportive […] [F17]; The people proved to be very supportive, as evidenced by the donations and various initiatives implemented for those most in need [F45].
- Empathy: I value the importance of professions that did not have a leading role before confinement, such as supermarket cashiers, carriers, cleaning staff […] [D22]; Seeing the working conditions and the work rhythms led me to empathize with the doctors, the nurses and all the professional figures who never stopped working [D27]; People empathize with those who suffer from the virus and with those who continue to work in this situation despite the adversities [F36].
- Responsibility: Be responsible and take the preventive measures indicated to us; social isolation [D23]; prevention, prudence, responsibility [D48].
- Value: Spending so much time without leaving home helps us learn to value things that previously seemed insignificant to us [D2]; I think about the waste of time that we sometimes do when we have so much free time that we don’t even know what to do and we stop being productive [D18]; that little value was given to supermarket employees and how important they are [D31]; Recognize the work that health personnel do daily and now more than ever [D39]; Value the difficult decisions that leaders and public health have to make [D45]; […] how important were the hugs, kisses and caresses that we don’t have now [D48].

This personal change shows that, after a while, a sense of well-being is self-perceived, which is achieved by the positive relationship and balance that exists between the actions carried out to adapt and overcome the situation and the personal appreciation of seeing that one is receiving: *I feel bad, I’ve lost everything [I23]; Since I have changed my habits, my well-being has improved […] [D17]; After 14 days confined and having established a routine, I spend my days better and I feel better [D28]; I have set goals for myself (exercise, household chores, university tasks, study…) everything organized in a schedule I feel better [D31]; Feeling good if I see how there are people who are overcoming COVID-19 and the curve is improving [F7]; I notice that, with everything I do, I am overcoming the situation and I feel good [F37].*

The results show that resilience acted as a guarantor between personal growth and the self-perception of well-being by favoring the process of continuous learning, adaptation, and subjective and personal evolution: *[…] adaptation to the situation, trying to grow [D17 ]; […] I see my adaptation while we are in this state of emergency [D23]; The need to cover free time at home and to keep the mind active began in new practices that quickly became a ritual: cooking with the family, exercising at home, reading […] [D47]; Instead of seeing it as a problem, I see it as an adaptation to the difficulties that this situation poses [F9]; The adaptation was necessary, new needs appeared and above all to be able to maintain mental health [F27].*

## 4. Discussion

This research provides relevant data on the process of the adaptation of the participating students to the traumatic situation they were experiencing (home confinement caused by COVID-19) and shows their evolution and personal growth. These results are supported by different investigations, which confirm that the different ways of coping with difficulties are part of the adaptive development of a human being [34,35].

The students developed coping strategies [34,36,37] and were able to perceive their well-being. Similar results to those obtained in other studies have showed that the participants developed a great ability to solve problems and achieve well-being [35].

A change is observed in the way of perceiving, feeling, and interpreting reality that is manifested through the appearance and/or improvement of personal skills such as self-esteem, self-knowledge, self-control, perseverance, and self-care. As with the results obtained in another study, these indicated a personal evolution [17]. It was noted that the participants felt uncertainty, fear, and anguish, and it was observed that, as the time of confinement passed, feelings of responsibility and care appeared, with participants referring to themselves and the people around them [38].

In some cases, interpersonal relationships with family and friends have changed, and the channel of interrelation has been modified, with social networks being an important point of support. It has been observed that relationships with family and friends are perceived as necessary, close, and revalued, which is further evidence of interpersonal growth and social evolution. All of them show the relevance of the interaction with other people who live the same reality, which causes mutual benefits, results in line with what was proposed in other research studies [18]. Moreover, the relevant role that social networks played during home confinement in communication with other people has been observed [39]).

Personal growth is achieved when there is a balance between the actions taken to deal with this situation and the achievement of previously established goals, which generates the perception of well-being, as identified in another study [20].

It is verified that, from negative experiences, positive consequences are extracted, such as solidarity, social conscience, perseverance, and empathy, among others. These may be the values that contribute to the acceptance and compliance with the restrictions imposed and therefore reduce the time of impact of the traumatic situation of home confinement. Traumatic situations affect people’s lives, and it is the resilience that favors them so that they can learn, grow, and feel good. This is in line with the findings of other studies [40].

This research study has some limitations. Firstly, the sample size is small, and it would be beneficial to include participants from all over Spain to strengthen the results of the study and may identify more variables involved. Secondly, the speeches of the students are conditioned by the degree of interest they have shown in participating in the research, and it was determined that some answers were more extensive, calm, and thoughtful compared to others that were shorter and more concise, although all valuable from a scientific point of view. In this sense, it would have been interesting to have been able to organize a discussion group with all the participants, once home confinement has ended, to delve into the topics under study. This has not been possible because the de-escalation phases of the confinement overlapped with the end of the course and the exam period. Thirdly, one of the future actions could be to carry out the research by expanding the study over time compared to pre- and post-pandemic.

Finally, carrying out quantitative studies to triangulate the results obtained, considering variables such as gender, age, etc., will broaden the knowledge about whether these variables may be influencing factors when dealing with conflict situations or crises through resilience. Moreover, information will be collected on the social and vital circumstances of the students to try to relate them to their emotions, feelings, and thoughts, and to be able to assess to what extent good or bad material and social conditions, from the start, are facilitators of personal growth, self-perception of well-being, and a perfect adaptation to adverse and negative situations.

## 5. Conclusions

The present research confirmed that resilience acts as a guarantor of personal growth and self-perception of well-being. This is the result of the voluntary, conscious, and intelligent evolution of the students, which was the object of the investigation. Moreover, personal changes, interpersonal changes, and changes in the philosophy of life are identified as factors of personal growth related to the situation and perception of well-being and health that this causes.

## Figures and Tables

**Table 1:** List of categories and citations.

Categories–Subcategories	Frequency	%
Personal growth	198	26.15
Personal changes	78	10.30
Changes interpersonal relationships	54	7.13
Changes in the philosophy of life	66	8.72
Wellness	79	10.43
Resilience	84	11.10
**TOTAL**	**757**	**100.00**

## Data Availability

The data supporting reported results of this article are available from the first author (paula.rios.dedeus@udc.es) in the Grupo de Investigación FORVI (Formación y Orientación para la Vida), Universidade da Coruña.
